# Harnessing the lymphatic system

**DOI:** 10.1007/s10741-024-10449-z

**Published:** 2024-10-15

**Authors:** Barbara Ponikowska, Marat Fudim, Gracjan Iwanek, Robert Zymliński, Jan Biegus

**Affiliations:** 1 Faculty of Medicine, Institute of Heart Diseases, Wroclaw Medical University, Borowska 213, Wroclaw, 50-556 Poland; 2 Duke University Medical Center, 10 Duke Medicine Cir, Durham, 27710-1000 USA; 3 Duke Clinical Research Institute, Duke University School of Medicine, 300 W Morgan St, Durham, 27701 USA

**Keywords:** Lymphatic system, Heart failure, Lymphatic imaging

## Abstract

Heart failure (HF) is a growing concern, with significant implications for mortality, morbidity, and economic sustainability. Traditionally viewed primarily as a hemodynamic disorder, recent insights have redefined HF as a complex systemic syndrome, emphasizing the importance of understanding its multifaceted pathophysiology. Fluid overload and congestion are central features of HF, often leading to clinical deterioration and hospital admissions, with the role of the lymphatic system previously largely overlooked, partly due to diagnostic challenges and visualization difficulties. With the advancement of those techniques, pathophysiological changes occurring in the lymphatic system during HF, such as enlargement of the thoracic duct and the increased lymphatic flow, are now becoming apparent. This emerging research has begun to uncover the interplay between lymphatic dysfunction and HF, suggesting novel therapeutic targets. Advances in molecular biology, such as targeting vascular endothelial growth factor and promoting lymphangiogenesis, hold promise for improving lymphatic function and mitigating HF complications. This article provides a comprehensive overview of the evolving landscape of lymphatic system-targeted therapies for HF. It explores various intervention levels, from mechanical lymphatic decongestion to pharmaceutical interactions and lymphatic micro-circulation, offering insights into future directions and potential clinical implications for HF management.

## Introduction

Heart failure (HF) is increasing in prevalence (~ 1–3% of the adult population) [1], has high mortality and morbidity, and is an unsustainable economic burden [2, 3]. Despite significant advancements in diagnostic and therapeutic modalities in HF, continued evaluation of its underlying pathophysiology is indispensable to developing novel, more effective therapeutic approaches. In the past, HF was perceived as a disorder primarily affecting the heart itself with accompanying changes in the cardiovascular system, and that is where research and clinical interest were placed [4]. However, more recently, we have witnessed a shift in the paradigm centred around the diseased heart and impaired haemodynamics toward HF being seen as a complex systemic syndrome, which better explains the progressive nature of the disease [4, 5].

Fluid overload, either due to gradual accumulation or rapid volume shifts, clinically manifesting as congestion, usually accompanied by elevation of natriuretic peptides, are cardinal features characterizing HF, and as such is featured in definitions of the disease in both the ESC and AHA HF guidelines [6–9]. Of note, during their journey along the natural history of the disease, patients with HF remain at constant risk of clinical deterioration with worsening signs/symptoms, which may ultimately require hospital admission [10]. The course of the disease is progressive but not linear, with worsening quality of life despite increasing levels of care [11]. The episodes of HF worsening always carry the risk of disease progression with poor outcomes and are typically driven by the development of uncontrolled congestion rather than by a low cardiac output [10, 12, 13]. Therefore, optimal control of water–ion homeostasis to achieve and maintain euvolemia and prevent episodes of congestion expansion is considered one of the fundamental elements of comprehensive HF management but remains a challenge in clinical practice [14–16].

**Fig. 1:**
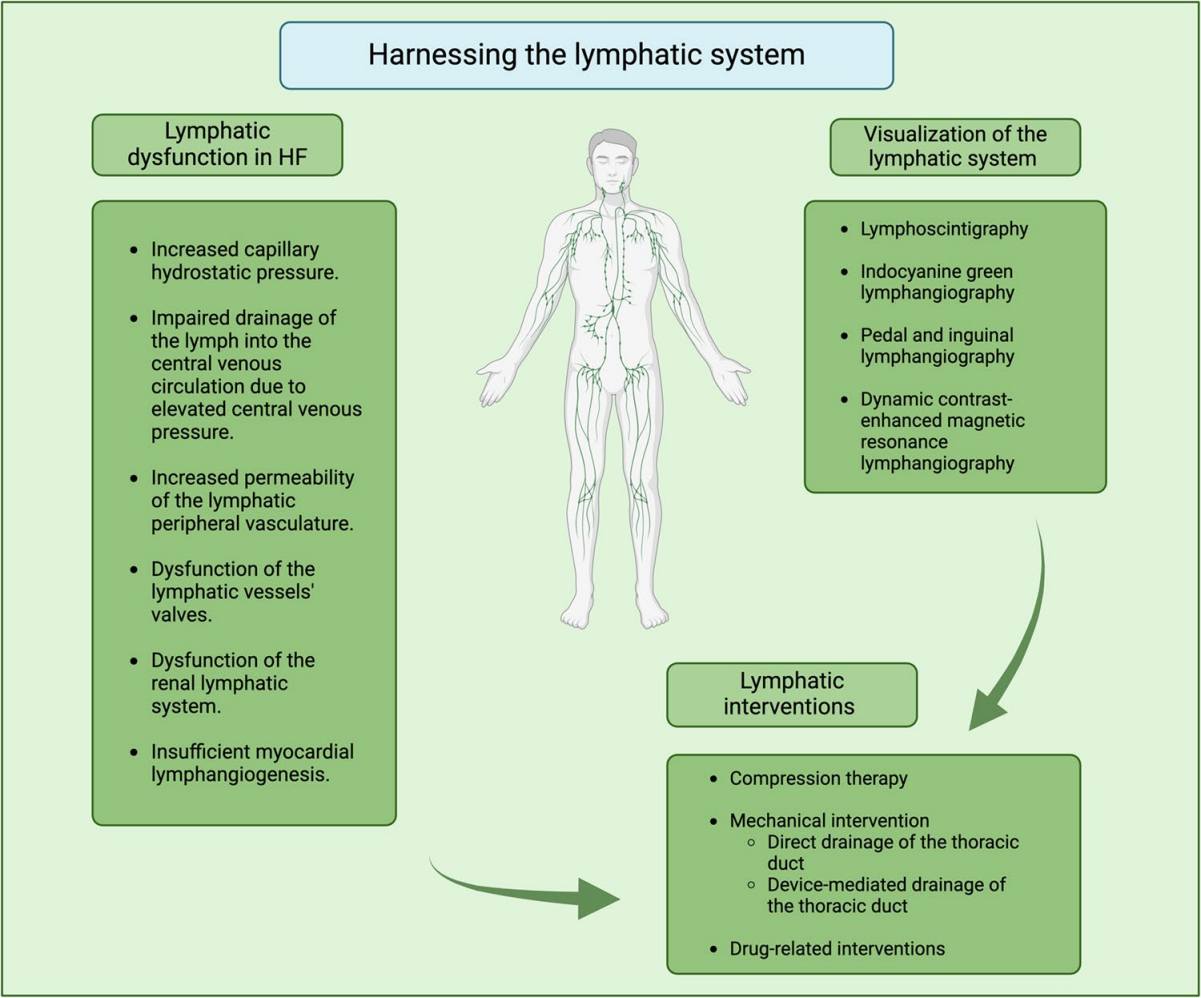
Graphic overview of the article contents. Created in BioRender

Only recently, in an attempt to optimize and propose new and effective decongestive HF strategies in ambulatory and in-hospital settings, attention has been turned toward the lymphatic system [1]. The primary role of the lymphatic system has been well-established and is the constant drainage of the filtered fluid from the interstitial space into the central venous system [17]. In normal homeostatic circumstances, the fluid production rate in the interstitial space matches the rate of lymph fluid return, preventing oedema in the peripheral tissues [17, 18]. Having in mind peripheral congestion as one of the cardinal features characterizing HF syndrome, intuitively, one may expect a significant role of the dysfunctional lymphatic system in HF. Surprisingly, this potentially relevant pathophysiological mechanism has been almost entirely abandoned and underestimated. This may at least partially be due to the challenges in assessing the lymphatic system, with a lack of simple diagnostic tools and the inherent complexity of visualization and access to the system itself. Nonetheless, recent studies have begun to unravel the complex interplay between lymphatic dysregulation and HF pathophysiology, shedding light on potential therapeutic targets [19]. Advances in molecular biology have opened promising avenues, such as targeting vascular endothelial growth factor (VEGF) and promoting lymphangiogenesis to improve lymphatic function and mitigate HF-associated complications [19]. By looking at different levels of potential intervention, from mechanical lymphatic decongestion through the interaction between pharmaceuticals and the lymphatic system, as well as targeting lymphatic micro-circulation, this article seeks to provide an overview of the evolving landscape of lymphatic system targeted therapies for HF, offering insights into future directions and potential clinical implications (Fig. 1).

## Brief overview of anatomy and physiology of the lymphatic system

The lymphatic system is an open, low-pressure circulatory system with no central pump, and the lymphatic capillaries collect the filtered fluid from the peripheral tissues, merge into lymphatic collectors, and connect to regional lymph nodes [1]. The collected fluid with immune cells, antigens, lipids, and proteomes generated by the metabolic activities of the tissues from the lymph is transported to the central venous system through two major lymphatic ducts: the right lymphatic duct and the thoracic duct, with the former draining lymph from the right side of the thorax, right arm, and right side of the head and neck and the latter from all the body except parts that are drained by the right lymphatic duct, and empties into the junction of the left internal jugular vein and the left subclavian vein [1, 20]. The lymphatic system returns 1–2 L of fluids to the central venous system per day and contains approximately 12 L of fluid at any point in time [1].

The characterization of lymph flow mechanics is beyond the scope of this paper (for more details, see references [1] and [20]. In brief, the peripheral lymphatic vessels exhibit specific cellular features to facilitate both the filtration process and the lymphatic flow [1, 21, 22]. The lymphatic capillaries are composed of a single layer of partly overlapping lymphatic endothelial cells with no basement membrane or pericytes; smooth muscle cells scantly cover the walls of pre-collectors with more smooth muscle cells in the collecting lymphatic vessels [1]. A lymphangion, which is a functional unit of the lymphatic vessel, has a pumping capacity regulated by preload, afterload, contraction frequency, and contractility, with pressures ranging from 20 to 120 mm Hg (resting-upright position), generated by lymphatic contraction [20, 23]. Of note, there are lymphatic valves in the system, and lymph flow is unidirectional in normal physiologic conditions [24]. Several external factors affect the lymph flow, namely intestinal peristalsis, respiratory intrathoracic pressure variations, contraction of the skeletal muscles, and the pulsation of neighbouring blood vessels [20].

The amount of filtered fluid across the semipermeable membrane of the blood capillaries is determined by the Starling equation and depends on three major factors: (a) the surface of the capillary area, (b) the difference between hydrostatic pressure in the capillaries and in the interstitium, and (c) the difference between oncotic pressure of the plasma protein and the of the interstitial protein [1, 20, 25]. It is important to remember that there is variability of the capillary hydrostatic pressures in different organs (e.g. under normal conditions, capillary hydrostatic pressure in soft tissue is around 35 mm Hg, whereas capillary pressure in normal pulmonary capillaries would be around 10 mm Hg) [20]. Similarly, there is considerable variability among different organs in interstitial oncotic pressure and protein concentration and permeability of the capillary wall to protein, which explains organ-specific differences in the composition and rate of lymph production [20].

## Visualization of the lymphatic system

To begin thinking about the lymphatic system in clinical terms, one must find a way of visualizing and assessing its function and structure. The inherent complexity of this task has presented an obstacle to popularizing the role of lymphatics in HF [1]. With readily accessible imaging modalities, clinicians would be able to assess patients’ lymphatic function in all stages of HF, monitor changes that might occur with disease progression, and offer pharmaceutical or mechanical interventions to alleviate symptoms. As the prevalence of HF increases [1] and the survival rate of HF patients continues to improve [26], a holistic approach to the disease management becomes even more important to enhance quality of life.

### Lymphoscintigraphy

Lymphoscintigraphy is an imaging technique that requires radiotracers, such as Tc-99 albumin solution [27], to be injected subcutaneously. The radiotracer is then absorbed into lymphatic vessels and gives off gamma rays, captured by a gamma camera and then turned into images of the lymphatic pathways. It is relatively minimally invasive, safe for patients with pulmonary dysfunction, and allows for visualization of deep lymphatics [28] and does not require specialized skills [27]. It is, however, less cost-effective than magnetic resonance; the results do not provide a detailed image of the lymphatic system, and the method does not allow for dynamic assessment of the lymphatic flow.

### Indocyanine green lymphangiography

One minimally invasive method is indocyanine green (ICG) lymphangiography. During the examination, which can be performed at the bedside, ICG dye is injected subcutaneously into the foot, after which the lymphatic flow is observed with a dedicated camera in real-time. The use of ICG lymphangiography in clinical practice is still limited and would require standardization in the assessment of what constitutes increased lymph flow rate; nonetheless, due to the method being relatively easy and quick to perform, it could be particularly useful in stratifying HF patients into impaired and non-impaired lymphatic flow groups on admission, to personalize treatment during hospitalization and potentially consider some of the interventions described further in this article. [1, 29, 30].

### Pedal and inguinal lymphangiography

Pedal lymphangiography (PL) is a method in which dye mixed with lidocaine is injected subcutaneously in between the toes. The lymphatic duct is then dissected and cannulated in both legs, and ethiodized oil followed by saline is injected to visualize the opacified cisterna chyli and the thoracic duct time. Pedal lymphangiography provides much more detailed results in comparison with ICG lymphangiography but is, in turn, time-consuming and technically challenging [31]. A less invasive alternative is intranodal lymphangiography (IL), which entails accessing the bilateral inguinal lymph nodes with a spinal needle under ultrasound guidance and injecting ethiodized oil until opacity is obtained at the L3 level of the abdominal lymphatics [31].

### Dynamic contrast-enhanced magnetic resonance lymphangiography

Dynamic contrast-enhanced magnetic resonance lymphangiography (DCMRL) constitutes the injection of gadolinium-based contrast into the inguinal nodes and the acquisition of MRI sequences to visualize the filling of lymphatic vessels until the thoracic duct’s entry into the venous angle is visible. While providing a detailed and dynamic three-dimensional volumetric measurement clarifies lymphatic flow, it is time-consuming, costly, and moderately invasive [32].

While various methods exist for visualizing and assessing the lymphatic system, each has its advantages and limitations. From ICG lymphangiography and lymphoscintigraphy being minimally invasive but less detailed and in the case of ICG lymphangiography limited to shallow lymphatic vessels assessment, through the more invasive PL, IL, and DCMRL, each technique offers insights into lymphatic function but also poses constraints regarding standardization, invasiveness, or cost. Additionally, the methods allow only partial visualization of the lymphatic system, excluding liver and intestinal lymphatics [33]. Therefore, developing new or enhancing existing techniques could be an important step in developing new therapeutic strategies and bringing lymphatics into clinical practice.

## Mechanisms of lymphatic congestion in heart failure

**Fig. 2:**
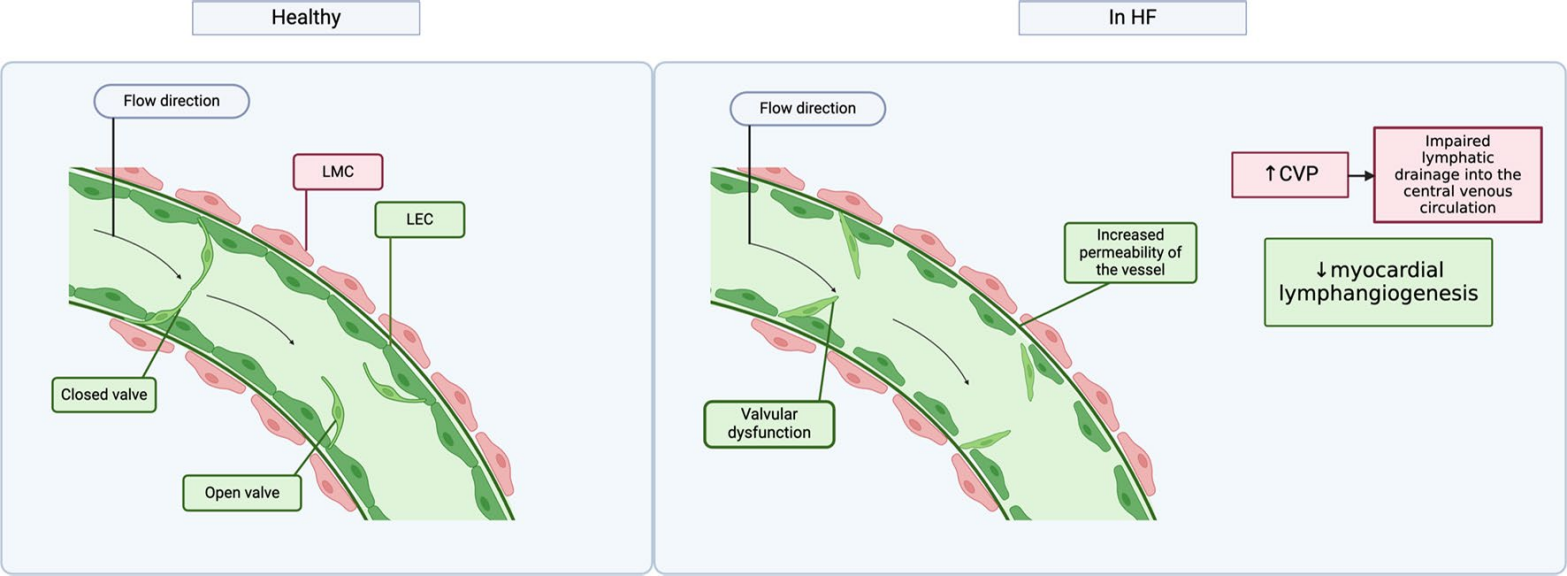
Lymphangion in a healthy state (left) and in HF (right). LMC, lymphatic muscle cell; LEC, lymphatic endothelial cell; CVP, central venous pressure. Created in BioRender

The lymphatic system is crucial for maintaining fluid homeostasis, and only in recent years has a growing body of evidence highlighted its profound impact on the clinical manifestations and progression of this condition [1, 20, 34, 35]. HF is characterized by systemic venous congestion, which underlies its clinical signs and symptoms, such as elevated jugular venous pressure, pleural effusions, hepatic enlargement, ascites, peripheral oedema, and dyspnoea [36]. A study performed by Damman et al. identified increased central venous pressure (CVP) as an independent driver of adverse outcomes and mortality [37]. Episodes of HF worsening are also driven mainly by fluid accumulation and venous congestion [10]. It appears that in parallel to the development of venous congestion, lymphatic drainage of the peripheral fluid is impaired in HF, which subsequently leads to lymphatic congestion, which potentiates the development of clinical signs/symptoms of HF [1, 20]. The following mechanisms may underlie lymphatic dysfunction and pathological accumulation of interstitial fluid in HF (Fig. 2) [1, 20]:

- *Increased capillary hydrostatic pressure.* According to the Starling fluid equation, increased capillary hydrostatic pressure leads to increased filtration in the peripheral tissues and, if not matched by lymphatic clearance, leads to fluid accumulation in the peripheral tissues.
- *Impaired drainage of the lymph into the central venous circulation due to elevated central venous pressure.* In the lymphatic system, CVP acts as the “afterload” for the thoracic duct drainage into the central circulation. Elevated central venous pressure impairs the decompression of the lymphatic system. Thus, effective diuresis reduces the lymphatic system afterload by decongesting the venous system*.*
- *Increased permeability of the lymphatic peripheral vasculature.* A proinflammatory state characterizing HF may lead to an increased vascular permeability due to disintegration of the vascular barrier, which facilitates a leak of large molecules into the interstitial space; of note, it increases a gradient of oncotic pressure between the intra- and extravascular space (favouring interstitial oncotic pressure) which further increases net fluid filtration; there are also some additional factors associated with the proinflammatory state which may potentially affect lymphatic vessels integrity and compliance [1], but their role in HF have not been studied.
- *Dysfunction of the lymphatic vessels’ valves.* As discussed above, lymphatic flow is unidirectional and regulated by the valves in the lymphatic vessels. Dysfunction of these valves can lead to lymphatic oedema, which is well-described in numerous chronic conditions associated with oedema in the peripheral tissues. Again, the role of dysfunctional lymphatic valves in HF has not yet been investigated.
- *Dysfunction of the renal lymphatic system*. A detailed discussion of dysregulated renal lymphatics in HF is presented elsewhere [34], in summary:

Elevated systemic venous pressure in HF leads to increased renal lymphatic pressure and increased capillary filtration, which is accompanied by sodium retention [38, 39]; moreover, increased capillary permeability (due to systemic proinflammatory activation and central haemodynamics) is an additional factor affecting renal lymph accumulation in HF; of note, within an encapsulated kidney, an increase of renal interstitial oedema may compromise lymphatic drainage which impairs renal function contributing to cardio-renal syndrome in HF [40].

Only recently, Boorsma et al. have suggested that renal lymphatic dysfunction in HF may interfere with returning of albumin (and other large molecules) to the central venous system, which manifests as albuminuria [41]; these authors found that albuminuria is related to congestion and is associated with an increased risk of mortality and hospitalizations for HF [41].

- *Insufficient myocardial lymphangiogenesis.* This can lead to myocardial interstitial fibrosis, cardiac remodelling, and dysfunction [42].

Although acting individually, the above-mentioned mechanisms are unlikely to cause accumulation of interstitial fluid considering efficient compensatory mechanisms. However, in HF, typically, they often occur simultaneously, impairing the system’s ability to compensate, resulting in lymphatic congestion and exacerbation of signs and symptoms of HF [1]. Moreover, it may be hypothesized that the observed dysfunction of capsulated organs (such as the liver and kidneys) during episodes of decompensation may, at least partly, be related to organ oedema and subsequent internal compression [40, 43–48].

## Lymphatic system in heart failure—current interventions and targets for future therapies

### Compression therapy

Compression therapy (CT) is a non-invasive treatment option, which includes the use of medical compression stockings, lower extremity compression wraps (LECW), manual lymph drainage, and active leg compression using pneumatic devices [49]. It is widely used in the management of chronic venous disease and lymphoedema [50], but despite its elemental role in the treatment of those diseases, it holds limited evidence in handling lower extremity oedema in HF patients [51]. Considering the lack of guidelines, some studies have been conducted to assess the efficacy of CT in HF. However, due to the heterogeneity of the patient population, diverse heart pathology, and varying degrees of clinical severity, it is still difficult to assess the validity of the existing evidence [49]. One study of 32 patients with acute decompensated HF looked at the efficacy of LECW in targeting decongestion, parallel to the use of diuretics. The results showed the LECW group to have a reduced hospital length of stay, fewer incidences of acute kidney injury, less continuous intravenous diuretic therapy, greater reduction in oedema, as well as improved quality of life as compared to the control group [51]. In a stable HF setting, another study was conducted, looking at 101 patients, most of them with heart failure with preserved ejection fraction (HFpEF), 86 of which continued CT for 1 month, showing decreased BNP levels and improved symptoms without any adverse events [52]. Some safety concerns regarding CT are, for one, the implications of mobilizing large amounts of blood from the lower extremity, resulting in volume overload and potentially transforming pre-existing pulmonary congestion into pulmonary oedema [53]. A small study by Wilputte et al. of 5 HF NYHA III and IV patients who underwent right heart catheterization to monitor the effects of multi-layered bandages on the lower limb and muscle contractions showed transient deterioration of right and left ventricular functions both with a rise in preload and afterload [54]. Patients did not suffer any sustained clinical impairment. However, the authors concluded that CT should not be used in cases of severe oedema due to unforeseen effects on the patient population [53]. CT is an easy to perform, non-invasive method, and available evidence suggests therapeutic potential in selected HF patient groups, with additional future research necessary to develop guidelines for its appropriate use.

### Mechanical intervention

One potential approach to achieving lymphatic decongestion is mechanical intervention (direct interventions on the lymphatic duct). Thus far, multiple studies have investigated this approach both in animals and humans. Notably, in a 1967 study on dogs with right-sided HF, thoracic duct-to-pulmonary vein shunts were created operatively to relieve the lymphatic system of excess interstitial fluid. The resulting lymphovenous anastomosis (LVA) proved successful in alleviating the signs and symptoms of congestion [55]. In 1991, a study of sheep with pleural effusions due to left atrial hypertension who underwent thoracic duct drainage was shown to cause a significant reduction in pulmonary oedema [56].

Human studies began with a paper published in 1963 by Dumont et al., which studied five patients with congestive HF [38]. These patients underwent thoracic duct cannulation to relieve lymphatic congestion, which the authors theorized was one of the underlying causes of the clinical manifestations of congestive HF—ascites, peripheral oedema, jugular venous distention, hepatomegaly, dyspnoea, and orthopnoea. During the procedure, they observed that the diameter of the thoracic duct was 2–4 times normal, with lymphatic flow 4–12 times normal. Immediately after cannulation, there was a significant improvement in the symptoms of HF, but after the lymphatic flow was reduced to normal, the symptoms returned [38]. In 1969, another study was carried out in which 12 HF patients underwent thoracic duct cannulation, with similar results both during and after cannulation (increased thoracic duct diameter, increased lymphatic flow). Patients who survived the 1-week post-drainage period noted a dramatic improvement in signs and symptoms [57]. One must, however, note that the mortality rate among the participants of the study was very high, which may reflect the fact that the studied population had advanced HF or other possibilities.

Direct drainage of the thoracic duct is associated with two major obstacles. The first is the risk of mechanical complications, including perforation of the thoracic duct and chylothorax. Chylothorax is associated with high mortality due to the nutritional and immunological loss it causes as well as fluctuating intravascular volumes [58]. Moreover, effective surgical repair is extremely difficult and rarely successful. Lymph escaping from the lymphatic system can also cause local damage that can be lethal. This brings us to the second caveat of this method. Given the nutritional and immunological roles of the chyle (lymph consists of fat, immunoglobulin, and lymphocyte), its prolonged drainage may have clinical implications similar to those of chylothorax and may be related to wasting.

In terms of recent approaches to mechanical intervention in the lymphatic system in the context of HF, the study by Abraham et al. is at the forefront [59]. As with attempts in the twentieth century, the authors targeted the interstitial space, this time using a device to create a low-pressure zone in the outflow area of the thoracic duct, thereby promoting increased lymphatic drainage. The device was first tested in sheep and showed beneficial effects in reducing pulmonary effusion, specifically reducing the amount of extravascular water in the lungs. It was then placed in a human HF patient with severe symptomatic HF. After 30 min of treatment in the catheter laboratory, the authors observed an increase in diuretic output, a reduction in creatinine and CVP, and a reduction in orthopnoea and oedema [59]. This concept is being tested in the first human DELTA-HF (The Safety and Feasibility of the eLym™ System for the Decongestion of Excess Lymphatic Fluid via the Thoracic Duct in Acute Decompensated Heart Failure) trial that recruits patients with acute heart failure (AHF) [60]. The eLym™ System, developed by WhiteSwell, is a minimally invasive catheter-based system targeting the excess interstitial fluid and aiding its drainage, placed in the left internal jugular and innominate veins near the left venous angle, where they connect to the thoracic duct. It works by creating a low-pressure zone to aid fluid drainage in addition to intravenous diuretics [60]. During the Heart Failure Society of America (HFSA) Annual Scientific Meeting 2023 in Cleveland, Ohio WhiteSwell presented initial results from the trial which thus far encompassed nine hospitalized patients who received eLym therapy in conjunction with diuretic therapy and a control group of six patients who received standard of care treatment with loop diuretics alone. Patients who underwent treatment with the eLym System plus loop diuretic lost a mean of 6.0 ± 4.6 kg from baseline to hospital discharge while maintaining kidney function, as measured by a stable or improved creatinine (mean Δ − 0.10 ± 0.12 mg/dL). The loop diuretic-only group lost a mean of 3.3 ± 3.7 kg. One treated patient (1/9, 11%) was hospitalized within 30 days of discharge [60]. This trial shows great promise for future use of device-based therapy.

In a 2024 retrospective study by Lee et al. [61], the authors looked at a population of 1400 patients who have undergone LVA as treatment for lymphoedema. In comparison with a selected group that has not undergone LVA, the LVA group showed an increased risk of developing HF, independent of cardiovascular risk factors and associated comorbidities [61]. This example demonstrates a need to investigate the possible lasting implications of mechanical interventions on the lymphatic system.

Device-based approaches to improve thoracic duct drainage and lymphatic flow have some benefits, as demonstrated in both animal and human studies, namely rapid and effective relief of symptoms that are often the reason for hospitalization in decompensated HF [57, 59, 60]. A potential limitation is the feasibility of these protocols in clinical practice, as the treatments are invasive and require specialized personnel and equipment. The procedures often require a radiologist or in some cases a surgeon trained in the specific type of intervention, in addition to the patient having to be closely monitored in a hospital setting in case of any adverse effects. Also, the long-term effects of the physical manoeuvres on the lymphatic and venous vessels and the longevity of the benefits shown should be further investigated, as the studies to date report an almost immediate return of symptoms after drainage termination [38]. For the time being, these interventions have not yet reached clinical practice and remain limited to research trials.

### Drug-related interactions with the lymphatic system

As previously mentioned, the lymphatic system proves a challenging target for therapy, as we still lack ways to comprehensively assess its function. The current diagnostic modalities focus mainly on lymphatic flow and its rate, often without engaging in assessing contractility. The functional units of the lymphatic system, lymphangions, connect to create chambers divided by unidirectional valves, propelling lymph by smooth muscle cell contractions, acting like a ‘primitive heart’ [62]. In fact, the lymphatic muscle shares electrophysiological properties with both vascular smooth muscle and cardiac muscle. Like vascular smooth muscle, lymphatic muscle cells respond to regulation by nitric oxide, prostaglandins, and histamine [22]. Lymphatic pumping is initiated in the smooth muscle cells by a pacemaker mechanism generating voltage-gated Ca^2+^ channel-induced action potentials, which supplies the depolarization and influx of Ca^2+^ necessary for the contractile mechanisms to induce the phasic constrictions of lymphangions and forward movement of lymph [22, 63]. These cardiovascular similarities can explain drug interactions and adverse effects on the lymphangion contractile function [64]. This physiological context allows us to investigate the connection between cardiovascular drugs and lymphatic function and possibly aid clinical practice.

Several available drugs can have stimulatory or inhibitory effects on lymphangion contractility but also on cardiovascular vessels. To clearly determine what are the drug’s effects, and on which vessels, either direct administration to the lymphatic system or assessment of non-lymphatic effects would be required. In a review by Russel et al., no FDA-approved drug unambiguously improved lymphatic contractility [64]. Currently, there are no medications specifically designed to directly target the lymphatic system in HF. Several potential mechanisms and medication targets could improve or interfere with the lymphatic system. However, certain molecules may serve as potential therapies for the lymphatic system in HF, such as [64]:

1. VEGF-C/VEGF-D agonists—to promote/modulate lymphangiogenesis and improve lymphatic drainage.
2. Inotropes (noradrenaline, dobutamine, levosimendan, etc.)—may support lymphatic contractility and flow.
3. Phosphodiesterase (PDE) inhibitors—which may enhance lymphatic vessel tone and contractility by affecting intracellular signaling pathways (e.g. PDE-4 inhibitors).
4. Endothelin receptor agonists—investigated for their ability to regulate lymphatic tone.
5. Anti-inflammatory agents (like corticosteroids, anakinra)—aimed at reducing lymphatic vessel damage and permeability caused by inflammation.
6. Immunomodulators—such as monoclonal antibodies, which may help regulate immune cell trafficking through the lymphatic system.
7. Prostaglandins—for their potential role in modulating lymphatic vessel permeability and contraction.
8. Antifibrotic agents—to prevent or reverse fibrosis within lymphatic tissues, which can occur in chronic inflammatory or fibrotic conditions.

In the future, targeted delivery of drugs to the lymphatic system can be achieved by formulating therapies using nanoparticle carriers, liposomes, or polymer-based systems, which enhance drug uptake by lymphatic vessels and ensure localized action within the lymphatic system. The review sets the foundation for potential future research on the exact effects and interactions of cardiovascular drugs and lymphatic function, which could be useful in combination with the proposed previous stratification of patients according to lymphatic activity and, as a result, a more personalized approach to pharmacological treatment.

### Micro-circulation

Lymphangiogenesis is typically triggered by tissue injury, inflammation, local endocrine drive, and mechanical stress [65]. In non-HF patients, lymphangiogenesis occurs as part of the body’s healing and remodelling process following events like myocardial infarction or other tissue damage. Key growth factors, including vascular endothelial growth factor C (VEGF-C), drive this process by promoting the growth and repair of lymphatic vessels [65].

In heart failure (HF) patients, however, lymphangiogenesis can become dysfunctional. Chronic inflammation, elevated venous pressure, ongoing neurohormonal overactivity, and persistent cardiac injury in HF may impair proper lymphatic vessel formation and function [66, 67]. As a result, the lymphatic system’s ability to clear interstitial fluid and remove inflammatory mediators becomes compromised, contributing to fluid retention, oedema, and further myocardial functional and histological abnormalities. Moreover, ongoing stress and inflammation can delay or disrupt this process, leading to insufficient or maladaptive lymphangiogenesis over time, which contributes to the HF progression. VEGF-C and vascular endothelial growth factor receptor 3 (VEGFR-3) have recently been researched in the context of both diagnostic and therapeutic tactics [68]. In a study done by Iwanek et al., serum VEGF-C levels were associated with signs and symptoms of congestion in patients with AHF, with an inverse relationship between the two. Additionally, a low level of serum VEGF-C was related to adverse clinical outcomes [69]. This presents the possibility of using VEGF-C to identify patients with an existing lymphatic dysfunction as part of HF and personalize treatment accordingly. Moreover, apart from its possible function as a biomarker, VEGF-3 and its receptor VEGFR-3 have recently been considered therapeutic targets.

## Conclusion

In summary, the lymphatic system’s role in HF pathogenesis has gained recognition, offering many potential targets for therapeutic intervention. The areas which require scientific focus are abundant, beginning with assessment and visualization through mechanical intervention and drug interactions with the lymphatic system. Additionally, targeting lymphangiogenesis, particularly through VEGF-C and its receptor VEGFR-3, presents therapeutic opportunities and potential biomarker application. Different strategies, when used in conjunction, can allow for a more personalized and, therefore, effective treatment plan. Overall, the lymphatic system offers a promising frontier in HF management.

## Declarations

Jan Biegus has received honoraria from Bayer, Boehringer Ingelheim, and AstraZeneca for lectures. He is also an associate Editor of the Heart Failure Reviews. Marat Fudim is a Guest Editor of Heart Failure Reviews.
